# Editorial

**Published:** 1906-09

**Authors:** 


### EDITORIAL.

The National Dental Association will meet in September in Atlanta, Ga. The question now rises—will it pay the dental profession, and especially those who have been always constant in their attendance at the National meetings? I think the dental profession, as a whole, and especially in the western section, has become so thoroughly disgusted with this constant harangue, politics and so forth, that they lose sight of the possible benefits that they might derive from the program.

The canvass for new President or for the one that it had already been promised to is full under way. There is probably some fear that he will not be elected as easily as the two previous ones, for so far as we know the canvass was not started until the meeting was reached in the two previous cases. This is certainly an interesting period in the history of the National Dental Association in so far as to not only involve the names of a few men in the profession, but it is a very serious proposition for the dental profession as a whole. There is no great or serious objection to those elected as officers, except that there nas been publicly made, charges against their character,and up to the present time,we have heard of no steps being taken for the purpose of vindicating their character or of removing the dirty stigma on the National Dental Association, or in other words, the body that represents the dental profession of this country.

When any of these men are interrogated on this matter, they say that Dr. Emory E. Bryant has made these charges against the officials of the National Dental Association. They say—why, that man iscrazy If it is true that he is demented, he ought to be placed where he could not defile the character and integrity of those gentlemen who are to represent the dental profession of the United States, but as we have just said,they don't seem to recognize the importanse of stopping such a clash as is going on.

..Furthermore, the amendment to the army and navy bill which would dispossess such men as John S. Marshall and Bob Oliver from the service at a time of life, when it is not an easy matter to again fall in practice, is certainly one of the most disgraceful things I have ever had brought under my observation, and yet we pretend to pose before the public eye as gentlemen of highest learning.

There is much that might be said on this subject,and that should be said now before the meeting of the Nationl Dental Association, because all of this is certainly of vast importance to the future of our profession.All that the officers of the National Dental Asociation need do is to come out and show Mr. National Dental Critic that he has got the wrong pig by the ear, and the the profession will stand up for the honest conduct of the Dental Society and its officers.—*American Dental Journal.*

